# Three broad classifications of acute respiratory failure etiologies based on regional ventilation and perfusion by electrical impedance tomography: a hypothesis-generating study

**DOI:** 10.1186/s13613-021-00921-6

**Published:** 2021-08-28

**Authors:** Huaiwu He, Yi Chi, Yun Long, Siyi Yuan, Rui Zhang, Yingying Yang, Inéz Frerichs, Knut Möller, Feng Fu, Zhanqi Zhao

**Affiliations:** 1 Department of Critical Care Medicine, State Key Laboratory of Complex Severe and Rare Diseases, Peking Union Medical College Hospital, Peking Union Medical College, Chinese Academy of Medical Sciences, Beijing, China; 2 Department of Anesthesiology and Intensive Care Medicine, University Medical Center of Schleswig-Holstein Campus Kiel, Kiel, Germany; 3 Institute of Technical Medicine, Furtwangen University, Villingen-Schwenningen, Germany; 4 Department of Biomedical Engineering, Fourth Military Medical University, 169 Changle Xi Rd, Xi’an, China

**Keywords:** Electrical impedance tomography, Lung ventilation, Lung perfusion, Acute respiratory failure, *V*/*Q*

## Abstract

**Background:**

The aim of this study was to validate whether regional ventilation and perfusion data measured by electrical impedance tomography (EIT) with saline bolus could discriminate three broad acute respiratory failure (ARF) etiologies.

**Methods:**

Perfusion image was generated from EIT-based impedance–time curves caused by 10 ml 10% NaCl injection during a respiratory hold. Ventilation image was captured before the breath holding period under regular mechanical ventilation. *DeadSpace*_*%*_, *Shunt*_*%*_ and *VQMatch*_*%*_ were calculated based on lung perfusion and ventilation images. Ventilation and perfusion maps were divided into four cross-quadrants (lower left and right, upper left and right). Regional distribution defects of each quadrant were scored as 0 (distribution% ≥ 15%), 1 (15% > distribution% ≥ 10%) and 2 (distribution% < 10%). Data percentile distributions in the control group and clinical simplicity were taken into consideration when defining the scores. Overall defect scores (*Defect*_*V*_, *Defect*_*Q*_ and *Defect*_*V*+*Q*_) were the sum of four cross-quadrants of the corresponding images.

**Results:**

A total of 108 ICU patients were prospectively included: 93 with ARF and 15 without as a control. PaO_2_/FiO_2_ was significantly correlated with *VQMatch*_*%*_ (*r* = 0.324, *P* = 0.001). Three broad etiologies of ARF were identified based on clinical judgment: pulmonary embolism-related disease (PED, *n* = 14); diffuse lung involvement disease (DLD, *n* = 21) and focal lung involvement disease (FLD, *n* = 58). The PED group had a significantly higher *DeadSpace*_*%*_ [40(24)% vs. 14(15)%, PED group vs. the rest of the subjects; median(interquartile range); *P* < 0.0001] and *Defect*_*Q*_ score than the other groups [1(1) vs. 0(1), PED vs. the rest; *P* < 0.0001]. The DLD group had a significantly lower *Defect*_*V*+*Q*_ score than the PED and FLD groups [0(1) vs. 2.5(2) vs. 3(3), DLD vs. PED vs. FLD; *P* < 0.0001]. The FLD group had a significantly higher *Defect*_*V*_ score than the other groups [2(2) vs. 0(1), FLD vs. the rest; *P* < 0.0001]. The area under the receiver operating characteristic (AUC) for using *DeadSpace*_%_ to identify PED was 0.894 in all ARF patients. The AUC for using the *Defect*_*V*+*Q*_ score to identify DLD was 0.893. The AUC for using the *Defect*_*V*_ score to identify FLD was 0.832.

**Conclusions:**

Our study showed that it was feasible to characterize three broad etiologies of ARF with EIT-based regional ventilation and perfusion. Further study is required to validate clinical applicability of this method.

*Trial registration* clinicaltrials, NCT04081142. Registered 9 September 2019—retrospectively registered, https://clinicaltrials.gov/show/NCT04081142.

**Supplementary Information:**

The online version contains supplementary material available at 10.1186/s13613-021-00921-6.

## Background

Acute respiratory failure (ARF) does not always present in conditions that are ideal for an immediate etiologic diagnosis [1]. Rapid and accurate identification of ARF etiology plays a critical role in the initial treatment of affected patients and is related to outcomes [2–4]. Physical examination and bedside chest radiography may be insufficient and lead to a delay in adequate patient management. The transportation of ARF patients for further examination with the aim of determining etiology might be associated with a high risk. Moreover, bedside lung–heart ultrasound also works as an acceptable tool to determine ARF etiology [5]. However, the abovementioned methods could not provide regional lung ventilation and perfusion information.

Electrical impedance tomography (EIT), a portable, noninvasive, radiation-free, bedside lung imaging method, has gained more attention in developing lung protective ventilation strategies for critically ill patients under mechanical ventilation [6, 7]. Recently, evidence has revealed that EIT has the potential to identify regional ventilation defects and make early diagnoses in ARF patients [8–10]. The saline contrast EIT method has been validated against electron beam CT imaging for assessing regional lung perfusions in several animals [11–15]. Recently, several studies demonstrated the clinical use of this method to detect regional lung perfusion in acute respiratory distress syndrome (ARDS) and acute pulmonary embolism (PE) patients in clinical practice [16–19].

Increasing clinical evidence has shown that the saline bolus-based EIT method might have the potential to identify various etiologies of acute respiratory failure in critically ill patients [20, 21]. We hypothesized that the regional ventilation and perfusion pattern by the contrast-based EIT method would be useful for a broad etiologic diagnosis of ARF. The aim of this study was to validate whether regional ventilation and perfusion data obtained by the saline-contrast EIT method could discriminate the three broad acute respiratory failure (ARF) etiologies (pulmonary embolism-related disease, diffuse lung involvement disease and focal lung involvement disease).

This study has been partially presented in a letter form in a previously reported study [16]. To further confirm the potential of using EIT to diagnose various ARF etiologies, a larger observational clinical study was conducted.

## Materials and methods

The study was approved by the Institutional Research and Ethics Committee of the Peking Union Medical College Hospital. Informed consent was obtained from all patients or next of kin before data were included in the study.

From May 2018 to July 2020, ICU patients with ARF or who had a new onset of ARF in the ICU were screened for eligibility when the research team was available. The diagnosis of ARF was based on the following criteria: arterial oxygen partial pressure to fractional inspired oxygen ratio, PaO_2_/FiO_2_ < 300 mmHg and/or peripheral oxygen saturation SpO_2_ < 94% under air condition and/or dyspnea [22, 23]. As the control group, postoperative ICU patients without ARF were included, since it was convenient and easy to implement saline bolus injection in these subjects. Only patients with central venous catheters were included for both groups. The exclusion criteria for the study patients were: age < 18 years, pregnancy, body mass index over 50 kg/m^2^, ribcage malformation, and any contraindication to the use of EIT (automatic implantable cardioverter defibrillator, chest wounds limiting electrode belt placement, implantable pumps, etc.). COVID-19 patients were not included in the present study, and our hospital was not designated for COVID-19 patients.

The clinical etiologic diagnosis of ARF was established in the hospitalization report using standardized tests [laboratory examination, chest X-ray, CT scan imaging and CT pulmonary angiography (CTPA), bedside ultrasound for lung and heart], and response to treatment assessed by the ICU team (described in Additional file 1: Table S1). Three broad etiological diagnoses of ARF were classified as follows: (1) pulmonary embolism-related disease (PED); (2) diffuse lung involvement disease (DLD), including diffuse interstitial syndrome, ground glass opacity and acute lung edema caused by fluid overload; and (3) focal lung involvement disease (FLD), including unilateral atelectasis/pneumonia (lobe or total), gravity-dependent consolidation (by chest CT and/or lung ultrasound), pneumothorax, mild/massive pleural effusion (parapneumonic effusion, empyema and hemothorax, etc.). Patients with multiple etiological diagnoses were classified by the attending physician to the main diagnosis based on the clinical examination and response to therapies. For example, a one-lung lobe resection patient enrolled in the ICU with new onset of dyspnea had PE and a small range of lung lesions. Since PE was the main factor contributing to ARF as diagnosed by the physician, the patient was classified into the PE group.

All the included patients underwent saline bolus EIT measurements in the supine position. Patient demographics and relevant clinical data were collected on the enrollment day. The following parameters were documented: age, sex, Acute Physiology and Chronic Health Evaluation II score (APACHEII) [24], heart rate, mean arterial pressure, PaO_2_, FiO_2_, SpO_2_, serum d-dimer and 28-day mortality as an outcome measure.

### Regional ventilation and perfusion measured by EIT

EIT measurements were performed with PulmoVista 500 (Dräger Medical, Lübeck, Germany). A silicone EIT belt with 16 surface electrodes was placed around the patient’s thorax at the 4th intercostal space level. All patients received standard care and no other research-related interventions were performed. EIT measurements were continuously recorded at 20 Hz when the patients were at relative stable condition after medical treatment. A bolus of 10 ml 10% NaCl was injected during a respiratory pause (for at least 8 s) through the central venous catheter. The respiratory pause was conducted via an end-expiratory hold maneuver with the ventilator in the intubated patients. The conscious patients were asked to hold their breath for 8–12 s at the end of expiration. The EIT data were digitally filtered using a low-pass filter with a cutoff frequency of 0.67 Hz to eliminate periodic cardiac-related impedance changes (for evaluation of both ventilation and perfusion). Perfusion evaluated via hypertonic saline bolus injection corresponded to a non-periodic impedance drop that was not influenced by low-pass filtering. Furthermore, the data were analyzed offline using customized software programmed with MATLAB R2015 (the MathWorks Inc., Natick, MA).

Regional ventilation map was calculated by subtracting the end-expiration from the end-inspiration image, which represents the EIT signal variation during tidal breathing. The tidal images before the apnea period were averaged to increase the signal-to-noise ratio:

1 $$V_{i} = \frac{1}{N}\sum\limits_{n = 1}^{N} {\left( {\Delta Z_{{i,{\text{Ins}},n}} - \Delta Z_{{i,{\text{Exp}},n}} } \right)} ,$$

where *V*_*i*_ is pixel *i* in the ventilation image; *N* is the number of breaths within the analyzed period; Δ*Z*_*i*,Ins_ and Δ*Z*_*i*,Exp_ are the pixel values in the raw EIT image at the end-inspiration and end-expiration, respectively.

Due to its high conductivity, 10% NaCl acts as an EIT contrast agent and passes through the pulmonary circulation thereby producing a dilution curve after bolus injection during the apnea period based on the first pass kinetics theory [25, 26]. Regional perfusion map was generated by the slope of regional impedance–time curves after saline bolus injection [27]. In brief, the regional impedance–time curves during the descending phase were fitted with linear regression:

2 $$\Delta Z_{i} (t) = a_{i} t + b,$$

where *t* is the time starting from one cardiac cycle after the initial descent in the global impedance curve caused by saline injection and ending at the trough of the global curve during the apnea period. Then the perfusion value of pixel *i P*_*i*_ in the perfusion image was equal to − *a*_*i*_. Furthermore, ventilated and perfused regions were defined as follows: region *k* is ventilated if:

3 $$V_{k} > 20\% \times \max (V_{K} )\quad K \in \left[ {1,1024} \right].$$

Similarly, region *g* is perfused if

4 $$P_{g} > 20\% \times \max (P_{G} ),\quad G \in \left[ {1,1024} \right].$$

Subsequently, three regions (number of pixels) were identified: regions that were only ventilated (*R*_V_), regions that were only perfused (*R*_P_) and regions that were both ventilated and perfused (*R*_V+P_). To correlate with clinical events, the following EIT-derived parameters were calculated:

5 $$DeadSpace_{\% } = R_{{\text{V}}} /\left( {R_{{\text{V}}} + R_{{\text{P}}} + R_{{{\text{V}} + {\text{P}}}} } \right) \times 100\% ;$$

6 $$Shunt_{\% } = R_{{\text{P}}} /\left( {R_{{\text{V}}} + R_{{\text{P}}} + R_{{{\text{V}} + {\text{P}}}} } \right) \times 100\% ;$$

7 $$VQMatch_{\% } = R_{{\text{V + P}}} /\left( {R_{{\text{V}}} + R_{{\text{P}}} + R_{{{\text{V}} + {\text{P}}}} } \right) \times 100\% .$$

For the quantitative analysis of the regional ventilation and perfusion distributions, the following terms were defined:

- Both ventilation and perfusion maps were divided into symmetrical, non-overlapping, four cross-quadrants: lower left (LL), lower right (LR), upper left (UL) and upper right (UR). Only the ventilated or perfused regions were evaluated. The regional ventilation distribution (%), perfusion distribution (%) and *V*/*Q* (%) were calculated in each quadrant (LL, LR, UL and UR).
- Distribution defects of each quadrant were scored as follows: 0 (quadrant distribution% ≥ 15%), 1 (15% > quadrant distribution% ≥ 10%) and 2 (quadrant distribution% < 10%). The quadrant distribution defect scores ranged from 0 (minimal) to 2 (maximal). The cutoff value of 15% was derived from the lowest 5th percentile of the quadrant distribution in the control group (including quadrant ventilation and perfusion distributions). Further ranges (between 10 and 15%, lower than 10%) were selected considering clinical simplicity and the frequency of ventilation and perfusion occurrence in all 744 quadrants in the study population of patients with ARF. The 5% ranges of these three score ranks were derived from the 50% gradient quadrants’ distributions from 5 to 25th percentile.
- *Defect*_*V*_, and *Defect*_*Q*_ scores were calculated by the sum of four cross-quadrants to denote the corresponding defect scores for ventilation and perfusion, respectively. *Defect*_*V*+*Q*_ was the combined score calculated as *Defect*_*V*_ + *Defect*_*Q*_.

Typical ventilation, perfusion and *VQ* matching images of one patient from the control group are shown in Fig. 4D.

### Statistical analysis

A descriptive analysis was performed. Normal distribution was assessed with the Kolmogorov–Smirnov normality test. Normally distributed results are presented as mean ± SD, whereas non-normally distributed results are presented as median (25th, 75th percentile). The Kruskal–Wallis test was used to compare groups on continuous variables, and Bonferroni correction was used to adjust the *P* value for multiple comparisons. Chi-square and Fisher’s exact tests were used to compare categorical variables where applicable. Comparisons of two continuous variables were performed using Spearman’s correlation and linear regression. Since there was no a priori information regarding the etiology clusters available prior to this study, the sample size estimation was only based on PE diagnosis. According to the standard PE diagnosis rate (clinical diagnosis and/or confirmed by computed tomography pulmonary angiography), we conservatively estimated that a sample size of 69 patients (including 62 patients without PE) would be required to detect a 1% error rate (*β* = 0.8, and *α* = 0.05) based on 80% specificity in a 10% prevalence of acute PE. The ability of EIT parameters to identify three preset etiologies (PED, DLD and FLD) was examined including both ARF and control patients. The areas under the receiver operating characteristic (AUC) curves were compared using a Hanley–McNeil test [28]. The cutoff values were selected to achieve higher specificities. If the specificity was > 90%, the cutoff value was chosen to reach the highest Youden’s index. Otherwise, the cutoff value was chosen to reach the highest specificity. All comparisons were two-tailed, and *P* < 0.05 was required to exclude the null hypothesis. The statistical analysis was performed using the software package SPSS 24.0 (SPSS Inc. Chicago, IL) and MedCalc 11.4.3.0 Software (Mariakerke, Belgium).

## Results

**Fig. 1:**
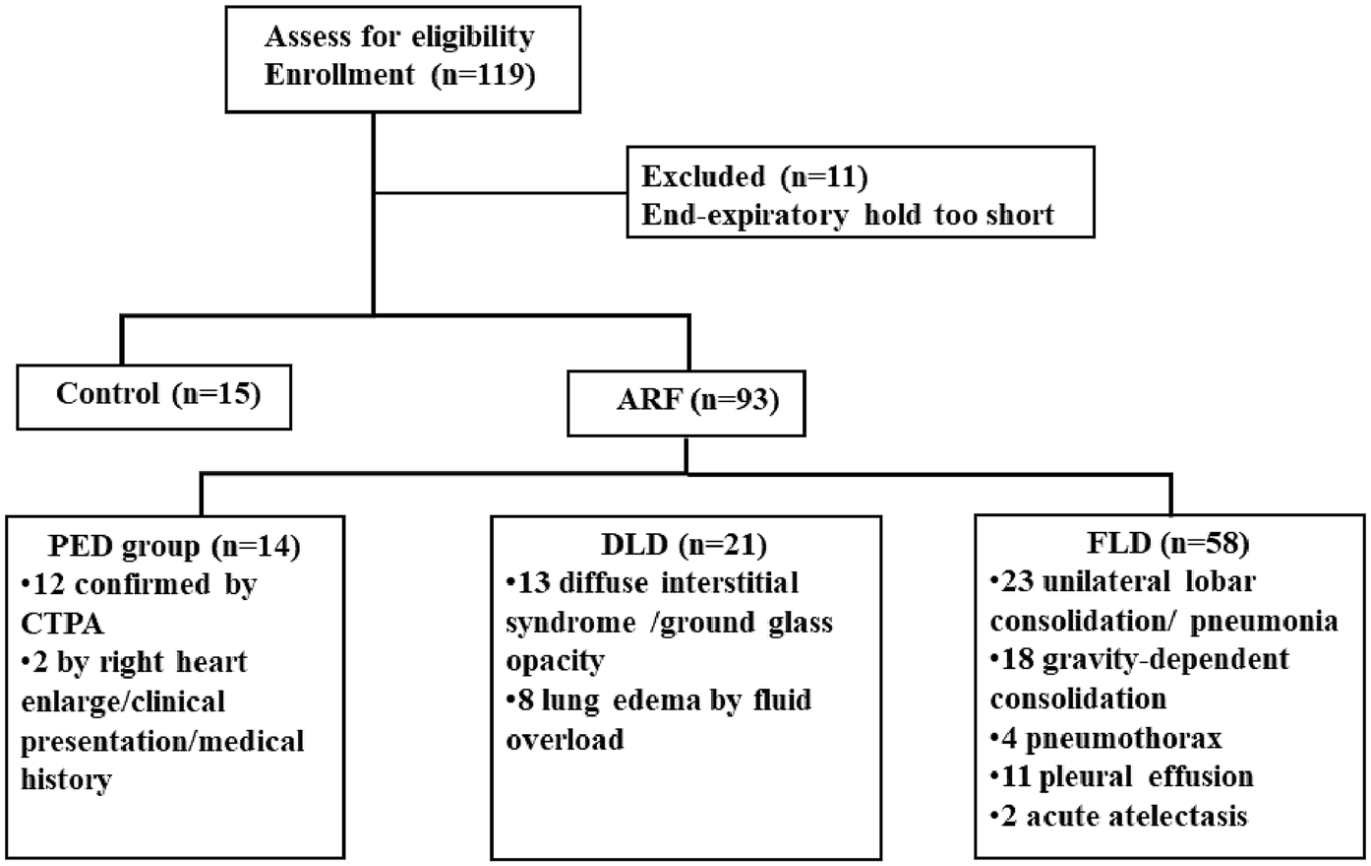
Flow chart of the enrolled patients. The control group was postoperative ICU patients without acute respiratory failure; ARF: acute respiratory failure; PED group: patients with pulmonary embolism-related disease; DLD group: patients with diffuse lung involvement disease; FLD group: patients with focal lung involvement disease; CTPA: CT pulmonary angiography

The flow chart of the enrolled patients is summarized in Fig. 1. A total of 93/104 ARF patients were analyzed (8/93 awake included 3 PED and 5 FLD and 84/93 sedated and intubated), and 11/104 ARF patients were excluded due to insufficient respiratory holding time with spontaneous breaths. Moreover, 15 sedated and mechanically ventilated patients without ARF admitted for postoperative monitoring (control group) were enrolled. The postoperative patients included in the study had no lung disease prior to the surgery and their oxygenation was normal before study inclusion. Significantly higher PaO_2_/FiO_2_ was found in the control group [390 (347, 474) vs. 210 (152, 276), *P* < 0.0001]. Moreover, the control group had a significantly higher *VQMatch*_*%*_ [72 (65, 80)% vs. 63 (47, 72)%, *P* = 0.002] and lower *Defect*_*V*_ [0 (0, 1) vs. 1 (0, 3), *P* = 0.003] and *Defect*_*Q*_ score [0 (0, 0) vs. 0 (0, 1), *P* = 0.01]. Demographics, clinical characteristics and EIT-related parameters of the study group and the control group are shown in Additional file 1: Table S2.

### Comparisons of different groups

**Fig. 2:**
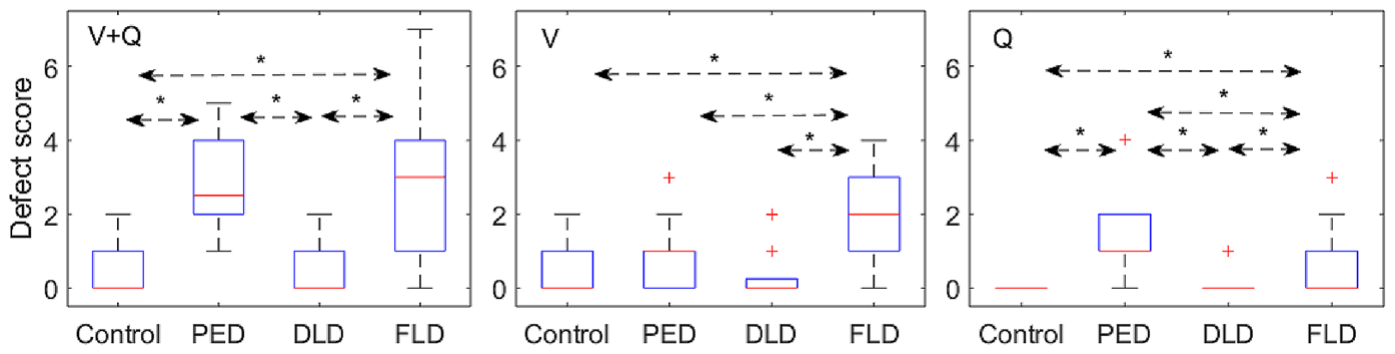
Comparisons of defect scores for ventilation (middle), perfusion (right) and combined (left) in the four groups. The control group was postoperative ICU patients without acute respiratory failure; PED group: patients with pulmonary embolism-related disease; DLD group: patients with diffuse lung involvement disease; FLD group: patients with focal lung involvement disease. The boxes mark the quartiles with median marked red, while the whiskers extend from the box out to the most extreme data value within 1.5 * the interquartile range of the sample. The red crosses are outliers. *P < 0.05

**Fig. 3:**
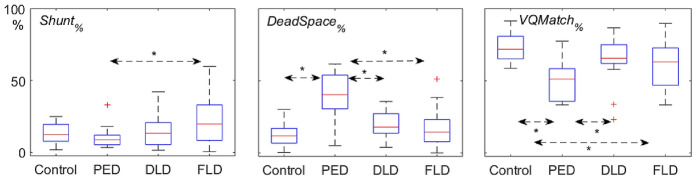
Comparisons of *Shunt*_*%*_, *DeadSpace*_*%*_, *VQMatch*_*%*_ in the four groups. The control group was postoperative ICU patients without acute respiratory failure; PED group: patients with pulmonary embolism-related disease; DLD group: patients with diffuse lung involvement disease; FLD group: patients with focal lung involvement disease. The boxes mark the quartiles with median marked red, while the whiskers extend from the box out to the most extreme data value within 1.5 * the interquartile range of the sample. The red crosses are outliers.*P < 0.05

Demographics, clinical characteristics of four groups (the control group and the three ARF subgroups: PED, DLD and FLD) are shown in Table 1. Differences in EIT-related parameters among the four groups are shown in Table 2. The PED group had a significantly higher *DeadSpace*_*%*_ and perfusion-defect score than the other groups (Figs. 2 and 3). The DLD group had significantly lower *Defect*_*Q*_ and *Defect*_*V*+*Q*_ score than the FLD and PED groups, and it had a similar *VQMatch*_*%*_, *Shunt*_%_, *Defect*_*V*_ and *Defect*_*Q*_ scores as the control group (Figs. 2 and 3). The FLD group had a significantly higher *Defect*_*V*_ score than the other groups (Fig. 2).

**Table 1:** Demographics, clinical characteristics in four groups

Variables	Control group *n* = 15	PED group *n* = 14	DLD group *n* = 21	FLD group *n* = 58	ANOVA-*P* value (Kruskal–Wallis test)
Age (years)	54 ± 14	59 (52, 68)	67 (55, 71)	65 (54, 75)	0.083
Sex (female/male)	6/9	10/4	9/12	21/37	N/A
APACHE II score	14 ± 5	15 (10, 17)	20 (15, 28)^a, b^	18 (14, 25)^a, b^	0.01
HR (bpm)	78 (65, 93)	87 (78, 93)	85 (80, 97)	94 (84, 103)^a^	0.019
MAP (mmHg)	87 ± 9	85 ± 10	84 ± 9	83 ± 12	0.336
FiO_2_ (%)	30 (30, 35)	45 (40, 80)^a^	40 (33, 58)^a^	40 (35, 75)^a^	< 0.0001
PaO_2_/FiO_2_ (mmHg)	390 (347, 474)	156 (86, 209)^a^	210 (117, 244)^a^	236 (170, 290)^a, b^	< 0.0001
d-Dimer (mg/L FEU)	1.0 (0.5, 3.0)	4.1 (2.7, 4.9)	4.6 (1.6, 6.2)	5.5 (1.9, 12.4)^a^	0.014
NE dose (μg/kg min)	0.00 (0.00, 0.09)	0.08 (0.00, 0.39)^a^	0.02 (0.00, 0.23)	0.08 (0.00, 0.26)	0.237
28-day mortality	0/15	4/14	3/21	11/58	N/A

The control group was postoperative ICU patients without acute respiratory failure; PED group: patients with pulmonary embolism-related disease; DLD group: patients with diffuse lung involvement disease; FLD group: patients with focal lung involvement disease. Normally distributed results are presented as mean ± SD, whereas non-normally distributed results are presented as median (25th, 75th percentile)

APACHE: Acute Physiology and Chronic Health Evaluation; HR: heart rate(bpm); MAP, mean arterial pressure (mmHg); PaO_2_: arterial partial pressure of oxygen; FiO_2_: fractional inspired oxygen concentration; N/A: not applicable

^a^*P* < 0.05 vs. control group (adjusted by the Bonferroni correction for multiple test)

^b^*P* < 0.05 vs. PED group (adjusted by the Bonferroni correction for multiple test)

^c^*P* < 0.05 vs. DLD group (adjusted by the Bonferroni correction for multiple test)

**Table 2:** Comparisons of EIT-related parameters in four groups

Variables	Control group *n* = 15	PED group *n* = 14	DLD group *n* = 21	FLD group *n* = 58	ANOVA-*P* value (Kruskal–Wallis test)
UR-ventilation (%)	31 ± 7	25 (16, 37)	29 (24, 34)	36 (23, 44)	0.088
UL-ventilation (%)	29 ± 9	24 (15, 33)	23 (19, 29)	28 (19, 39)^b^	0.206
LR-ventilation (%)	22 ± 10	23 (19, 31)	23 (19, 29)	18 (10, 26)	0.041
LL-ventilation (%)	17 ± 6	28 (19, 30)	22 (16, 25)	18 (8, 25)^b^	0.010
UR-perfusion (%)	24 (19, 30)	20 (9, 34)	23 ± 6	26 ± 11	0.271
UL-perfusion (%)	27 ± 5	22 (17, 27)	26 (21, 30)	27 (21, 33)	0.200
LR-perfusion (%)	22 (21, 27)	18 (8, 30)	25 (21, 29)	21 (15, 27)	0.147
LL-perfusion (%)	22 (21, 30)	37 (26, 42)	22 (19, 27)	22 (17, 29)^b^	0.015
UR-*V*/*Q* (%)	1.3 (1.1, 1.5)	1.3 (0.9, 1.7)	1.2 (1.0, 1.5)	1.2 (1, 1.7)	0.978
UL-*V*/*Q* (%)	1.1 (0.8, 1.3)	0.9 (0.5, 1.8)	1.0 (0.7, 1.2)	1.1 (0.8, 1.3)	0.795
LR-*V*/*Q* (%)	0.9 (0.7, 1.1)	1.5 (1.0, 2.6)	1.1 (0.9, 1.2)	0.8 (0.6, 1.1)^b^	0.09
LL-*V*/*Q* (%)	0.6 (0.7, 0.8)	0.8 (0.6, 1.0)	0.7 (0.7, 1.1)	0.7 (0.4, 1.0)	0.250
*VQ Match*_*%*_	72 (65, 80)	51 (36, 58)^a^	66 (62, 75)^b^	63 (47, 73)^a^	< 0.0001
*DeadSpace*_*%*_	12 (7, 17)	40 (30, 54)^a^	18 (14, 27)^b^	14 (8, 23)^b^	< 0.0001
*Shunt*_*%*_	12 (8, 19)	9 (5, 12)	13 (5, 20)	20 (8, 33)^b^	0.015
*Defect*_*V*_ score	0 (0, 1)	1 (0, 1)	0 (0, 0.5)	2 (1, 3)^a, b, c^	< 0.0001
*Defect*_*Q*_ score	0 (0, 0)	1 (1, 2)^a^	0 (0, 0)^b^	0 (0, 1)^a, b, c^	< 0.0001
*Defect*_*V*+*Q*_ score	0 (0, 1)	2.5 (2, 4)^a^	0 (0, 1)^b^	3 (1, 4)^a, c^	< 0.0001

The control group was postoperative ICU patients without acute respiratory failure; PED group: patients with pulmonary embolism-related disease; DLD group: patients with diffuse lung involvement disease; FLD group: patients with focal lung involvement disease. Normally distributed results are presented as mean ± SD, whereas non-normally distributed results are presented as median (25th, 75th percentile)

UR: upper right; UL: upper left; LR: lower right; LL: lower left; *V*/*Q* (%): relative regional ventilation/corresponding regional perfusion, both in percentage

^a^*P* < 0.05 vs. control group (adjusted with the Bonferroni correction for multiple test)

^b^*P* < 0.05 vs. PED group (adjusted with the Bonferroni correction for multiple test)

^c^*P* < 0.05 vs. DLD group (adjusted with the Bonferroni correction for multiple test)

### Correlation between PaO_2_/FiO_2_ and EIT-parameters

PaO_2_/FiO_2_ was significantly correlated with *VQMatch*_*%*_ (*r* = 0.324, *P* = 0.001), *DeadSpace*_*%*_ (*r* = − 0.301, *P* = 0.002) and *Defect*_*Q*_ score (*r* = − 0.212, *P* = 0.027). Regional ventilation, perfusion distributions and the corresponding *V*/*Q* (%) ratio were not significantly correlated with PaO_2_/FiO_2_.

### Diagnostic ability of EIT parameters for determining the three preset etiologies (PED, DLD and FLD) in ARF patients

The PED patients (14/93) had a significantly higher *DeadSpace*_*%*_, *Defect*_*Q*_ score, and LR-*V*/*Q* (%) and lower *VQMatch*_%_ and *Shunt*_%_ than non-PED patients. EIT-related parameters have a significantly higher performance regarding the diagnosis of PE than d-dimer. *DeadSpace*_*%*_ resulted in the highest AUC. A cutoff value of *DeadSpace*_*%*_ > 30.37% was used for the diagnosis PED, resulting in a sensitivity of 78.6% and a specificity of 92.4% (Table 3).

**Table 3:** ROC areas and cutoff value of parameters for identifying PE, DLD and FLD in 93 ARF patients

Items	Parameters used	ROC area	95% CI	Cutoff value	Sensitivity (%)	Specificity (%)
Identification of PED (14/93 PED)	*DeadSpace*_*%*_	0.894^a^	0.821–0.945	> 30.37%	78.6	92.4
	*Defect*_*Q*_ score	0.787^a^	0.672–0.902	> 3.5	21.4	100
	*VQ Match*_*%*_	0.752^a^	0.624–0.885	< 36.6%	35.7	96.2
	*Shunt*_*%*_	0.697^a^	0.575–0.819	< 3.23%	7.1	91.1
	LR-*V*/*Q* (%)	0.750^a^	0.577–0.923	> 1.72	50	94.9
	d-Dimer (mg/L FEU)	0.457	0.328–0.586	> 6.1	14.3	60.8
Identification of DLD (21/93 DLD)	*Defect*_*V*_ score	0.837	0.754–0.920	< 0.5	76.2	79.2
	*Defect*_*Q*_ score	0.726	0.621–0.832	< 0.5	85.7	55.6
	*Defect*_*V*+*Q*_ score	0.893	0.828–0.959	< 0.5	66.7	90.3
Identification of FLD (58/93 FLD)	*Defect*_*V*_ score	0.832	0.750–0.915	> 2.5	39.7	97.1
	*Defect*_*V*+*Q*_ score	0.745	0.644–0.846	> 4.5	20.7	97.1
	*Shunt*_*%*_	0.674	0.565–0.783	> 29.3%	34.5	91.4
	*DeadSpace*_*%*_	0.728	0.621–0.835	< 7.9%	25.9	91.4

PED: pulmonary embolism-related disease; DLD: diffuse lung involvement disease; FLD: focal lung involvement disease; ARF: acute respiratory failure; ROC: receiver operating characteristic; CI: confidence interval; LR-*V*/*Q* (%): relative regional ventilation/corresponding regional perfusion of the lower right region of interest

^a^*P* < 0.05 vs. d-dimer

The DLD group (21/93) had a significantly lower *Defect*_*V*_, *Defect*_*Q*_, and *Defect*_*V*+*Q*_ scores than the non-DLD groups. The AUC for using *Defect*_*V*+*Q*_ to diagnose DLD was 0.893. The cutoff of *Defect*_*V*+*Q*_ was < 0.5 for the diagnosis of DLD, resulting in a sensitivity of 66.7% and a specificity of 90.3% (Table 3).

The FLD group (58/93) had a significant higher *Defect*_*V*_, *Defect*_*V*+*Q*_ scores, *Shunt*_%_ and lower *DeadSpace*_*%*_ than non-DLD patients. The AUC for using *Defect*_*V*_ to diagnose FLD was 0.844. The cutoff of *Defect*_*V*_ was > 2.5 for the diagnosis of FLD, resulting in a sensitivity of 39.7% and a specificity of 97.1% (Table 3).

### Phenotype of EIT ventilation/perfusion images for the three broad ARF etiologies

**Fig. 4:**
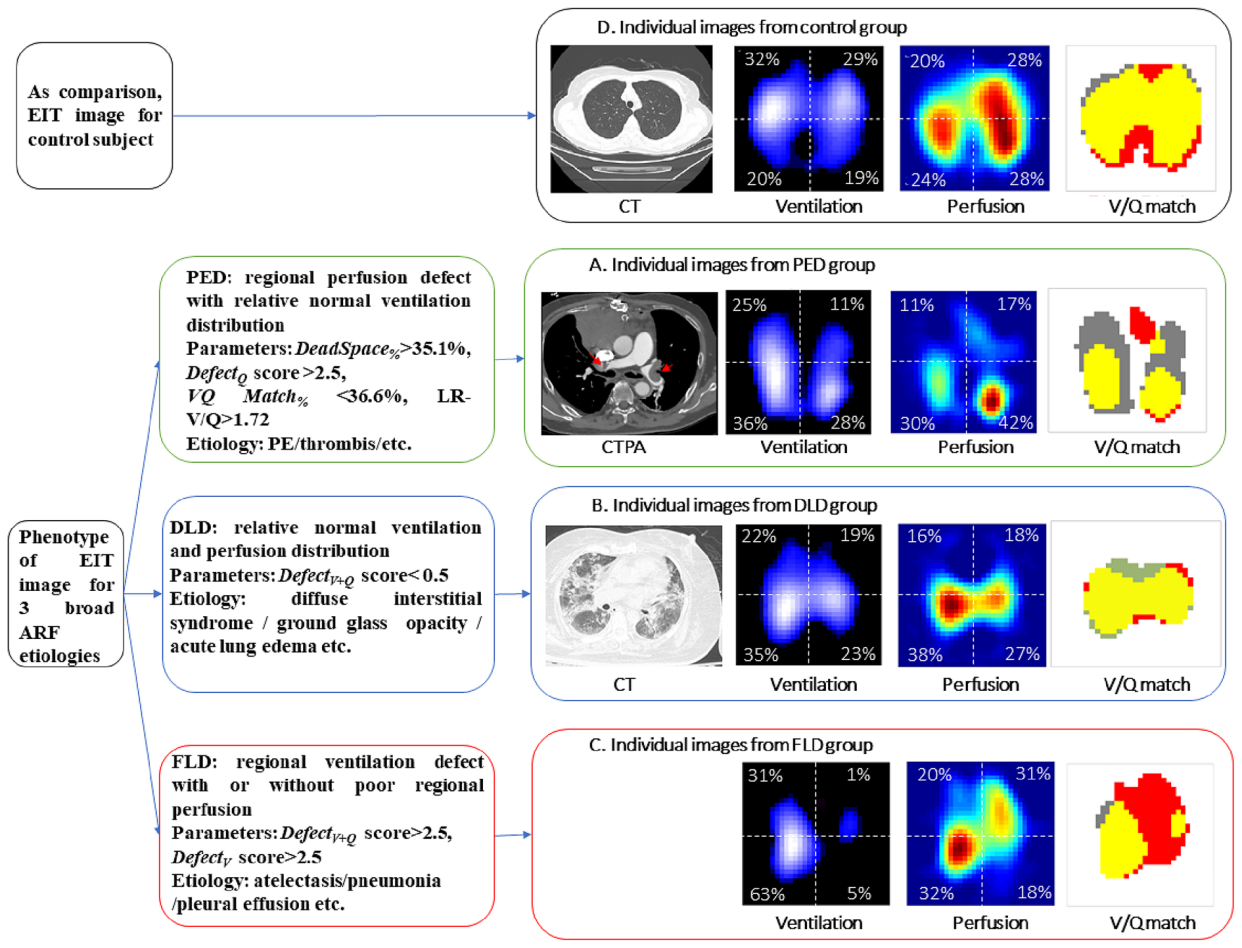
Phenotype of ventilation/perfusion and individual images by EIT for the three broad ARF etiologies. The control group was postoperative ICU patients without acute respiratory failure; PED group: patients with pulmonary embolism-related disease; DLD group: patients with diffuse lung involvement disease; FLD group: patients with focal lung involvement disease. **A** Patient from the PED group (CTPA: demonstrated large embolism in both left and right main pulmonary arteries. Ventilation image: upper right (UR) 25%, upper left (UL) 11%, lower right (LR) 36%, lower left (LL) 28% (% denoted the ventilation distribution), *Defect*_*V*_ score was 1. Low ventilated regions are marked in dark blue and high ventilated regions in light blue to white. Perfusion image: upper right (UR) 11%, upper left (UL) 17%, lower right (LR) 30%, lower left (LL) 43%, *Defect*_*Q*_ score was 1. Regions with high perfusion are marked in red and low perfusion in green. *V*/*Q* match image: percentage of *Shunt*_*%*_ area in red was 10% of the lung regions, *DeadSpace*_*%*_ area in grey 46%, and *VQMatch*_*%*_ region in yellow 43%. (partially adapted from our recent case report [47]). **B** A patient with ground glass opacity from the DLD group (CT: diffuse opacities in both lungs. Ventilation image: UR 2%, UL 19%, LR 35%, LL 23%, *Defect*_*V*_ score was 0. Perfusion image: UR 16%, UL 18%, LR 38%, LL 27%, *Defect*_*Q*_ score was 0. *V*/*Q* match image: *Shunt*_*%*_ 6%, *DeadSpace*_*%*_ 15%, and *VQMatch*_*%*_ 78% (partially adapted from our previous study with permission of the American Thoracic Society. Copyright © 2020 American Thoracic Society [16]. All rights reserved). **C** A patient with acute left lung atelectasis from the FLD group (ventilation image: UR 31%, UL 1%, LR 63%, LL 5%, ventilation-defect score was 4. Perfusion image: UR 20%, UL 31%, LR32%, LL 18%, *Defect*_*Q*_ score was 0. *V*/*Q* match image: *Shunt*_*%*_ 55%, *DeadSpace*_*%*_ 3%, and *VQMatch*_*%*_ 42%). **D** A patient from the control group. Percentage of intrapulmonary shunt area in red was 17%, dead-space fraction area in grey 5%, and *VQMatch*_*%*_ region in yellow 78%. *Defect*_*V*_ and *Defect*_*Q*_ scores were 0

These related characteristic EIT parameters that produced specificities > 90% of ventilation/perfusion phenotypes were retained for three broad ARF etiologies (Fig. 4). Moreover, typical ventilation, perfusion, VQ matching images and CT images of individual patient subgroups are shown in Fig. 4. Defects of regional perfusion with normal ventilation were observed in the PED patient (Fig. 4A). No cognizable defect of regional ventilation and perfusion was observed in the DLD patient (Fig. 4B). Defects of regional ventilation with relatively normal regional perfusion in left lung were found in FLD patients with acute left lung atelectasis (Fig. 4C).

## Discussion

The present study found that regional ventilation and perfusion measured by the contrast based EIT method were able to characterize three ARF etiologies (PED, DLD, FLD) in critically ill patients at the bedside. To the best of our knowledge, the present study was the largest sample and first clinical study of using the saline-based EIT method to delineate various pathologies of ARF.

Lung EIT as a functional imaging method has demonstrated its capability of clinical use in individual PEEP titration and other ventilator parameter settings at the bedside [29–32]. Several EIT applications in identifying etiologies of ARF (pleural effusion, pneumothorax and PE etc.) and assess the effect of PEEP on regional *V*/*Q* have been reported in animal and clinical studies [8–10, 16, 19, 27, 33–35]. In the present study, we used functional ventilation and perfusion distribution by EIT to characterize the three types of ARF etiologies.

### EIT for PED

Previous experimental studies have shown that regional lung perfusion after pulmonary embolism-like events could be effectively detected by EIT and bolus saline [12, 14, 34]. Recently, several clinical cases reported using EIT and bolus saline to detect PE in clinical practice [16, 18, 19, 36]. Our data supported the conclusion that the *DeadSpace*_*%*_ and *Defect*_*Q*_ score had a significantly better performance than d-dimer for PE diagnosis (Table 3). A high d-dimer concentration was considered an indication for anticoagulation treatment in ARF patients with suspected PE [37, 38]. Since many factors such as operation, tumor, and inflammation could also independently cause an increase in d-dimer, it is not surprising that d-dimer did not show a good ability to diagnose PE in the mixed patient population. Further study is required to examine whether EIT-based PE diagnosis could change clinical decisions and limit unnecessary anticoagulation in a critical condition, especially pertaining to postoperative patients.

Moreover, a high *DeadSpace*_*%*_ could be observed in severe ARDS patients, which might result from lung microvascular embolism. Marui et al. used a similar EIT method to show an elevated ventilation–perfusion mismatch, with a larger prevalence of ventilated nonperfused lung units (dead space) in comparison to perfused nonventilated units (shunt) in COVID-19 patients with ARDS [17]. This condition was not taken into consideration in the present study. Here, we emphasized the potential advantage of using EIT to determine submassive/massive PE in critically ill patients with heavy devices and a high risk of transfer for CTPA examination.

### EIT for DLD

There are a wide variety of causes of DLD in ICU patients, which are typically diagnosed with chest radiography, CT, and ultrasound [39]. In the present study, DLD of ARF was classified as diffuse pulmonary alveolar/interstitial/lobular disease without a significant focal lesion. Since the damage mainly affects the alveoli and interstitial tissue in a diffuse manner and spatial resolution of EIT is limited, an obvious defect of regional ventilation and perfusion is not expected to be found in the DLD patients, which is in harmony with and explains our results (Table 2). The DLD group had a low *Defect*_*V*_ and *Defect*_*Q*_ scores, which were similar to the control group. We presented a concept of low *Defect*_*V*+*Q*_ score that supports DLD diagnosis in the primary assessment of ARF patients. The AUC for using a low *Defect*_*V*+*Q*_ score (< 0.5) to diagnose DLD was relatively high (0.893). Moreover, estimation of extravascular lung water could be useful for further differential diagnosis in DLD patients. Quantitative evaluation of extravascular lung water by EIT has been reported in human and animal studies [21, 40]. Further studies with larger samples of DLD should be conducted to examine the efficacy of EIT-based parameters (including extravascular lung water assessment) on DLD subcategory classification.

### EIT for FLD

EIT has been proven to effectively measure ventilation defects by impedance monitoring in different pathophysiologic conditions, such as pleural effusion, pneumothorax, pneumonia, consolidation, atelectasis etc. The present study also supported that EIT could detect regional ventilation defects caused by various causes. Both extrapulmonary (pneumothorax and pleural effusion) and intrapulmonary (atelectasis/pneumonia/consolidation) lesions were included in the FLD. As expected, the highest *Defect*_*V*_ scores were found in the FLD group compared to the other groups. The cutoff *Defect*_*V*_ score was > 2.5 for the diagnosis of FLD, resulting in a sensitivity of 39.7% and a specificity of 97.1% in the present study (Table 3). Moreover, the regional perfusion varied in the ventilation-defect quadrants. Studies have suggested that regional perfusion may increase in damaged lung areas in pneumonia and ARDS [29, 41] but could also fall in the injured regions due to obstruction or compression of pulmonary capillaries [42, 43]. A recent animal study found experimental atelectasis with minimal tidal recruitment/derecruitment, and mechanically inspiratory breaths redistributed blood volume away from well-ventilated areas [44].

### Clinical implications and suggested algorithm

The greatest contribution of our study is that the phenotype of ventilation/perfusion images and related parameters from EIT were determined for three broad ARF etiologies. Moreover, these results were consistent with the pathophysiologic mechanism. Someone might argue that these related parameters had high specificities > 90% but relatively low sensitivities (< 80%). Several potential reasons may be considered: functional EIT measurement of ventilation and perfusion does not completely reflect anatomical location of ventilation and perfusion changes. Small lung lesions might not cause a significant change in regional perfusion or ventilation. Functional changes in ventilation and perfusion are complicated and variable in different lung lesions. Correlation between PaO_2_/FiO_2_ and EIT parameters were weak, which coincided with a recent study from Spinelli et al. [45]. They found a weak negative correlation between the percentage of only perfused units and PaO_2_/FiO_2_ ratio (*r* = − 0.293, *P* = 0.039) in ARDS patients. We suspect that with limited spatial resolution, EIT might not be able to capture small *V*/*Q* mismatch. Besides, *V*/*Q* mismatch does not account for 100% the decrease of PaO_2_/FiO_2_. Ventilation and perfusion could be both significantly decreased and still matched. The PaO_2_/FiO_2_ would not be satisfactory in this case.

Because of the limitation of using functional ventilation/perfusion defects to reflect the ARF etiologies of anatomical location, we focused on the diagnostic specificity of EIT images. The described EIT method should be taken as the preliminary assessment for broad ARF etiologies but not for precise etiology identification. We stressed that these significant signs (high *DeadSpace*_*%*_, *Defect*_*V*_ and *Defect*_*Q*_ scores) were related to the potential etiologies of ARF. These related parameters could work as feature indicators for the identification of broad etiologies of ARF and provide a probable diagnostic direction at bedside. Moreover, the relatively normal ventilation and perfusion distribution should be interpreted with caution in ARF patients. Both DLD and small lung lesions could have a “normal” distribution of ventilation and perfusion. Our proposed method has potential advantages in the differential diagnosis of life-threatening ARF, especially for unstable patients who require bedside monitoring tools. A scoring system for ventilation and perfusion distribution defects was defined in the present study. There were several potential advantages of the scoring system: (1) the scoring system is feasible and convenient at the bedside. (2) Regional ventilation and perfusion data from EIT are complex and often hard to interpret. For examples, *Deadspace*_*%*_ can categorize PE patients but no other etiologies. With the regional ventilation and perfusion images, it is hard to identify DLD directly. Hence, from the perspective of overall evaluation, the proposed scoring system has the potential to identify the predefined three ARF etiologies, which was supported by the present results.

### Limitations and future prospect

This work has some limitations. First, our study was carried out in a single center, and a small number of patients were included. The primary aim of study was to investigate the relationship of oxygenation and regional ventilation and perfusion assessed by the indicator based EIT method. The hypothesis of three broad ARF etiologies was generated with some of the results and analysis done. Second, the three broad etiologic diagnoses of ARF were established in a combined assessment by the ICU team based on an objective examination and standards (Additional file 1: Table S1). Strict predefined criteria of CT scan for the ARF etiology was lacking. Unfortunately, a validation for all the study subjects using radiography (e.g., CTPA and CT) was ethically impossible based on the study design. PE might also have been present in patients with DLD or FLD. The potential confounders would need to be considered when interpreting the results. Nevertheless, the proposed EIT method was used to diagnose broad ARF etiologies and not for precise and comprehensive diagnoses. Although the investigators were not totally blinded to the patient’s clinical presentation, the EIT profiles were established based on objective data. Third, only one main ARF etiology was identified based on a comprehensive clinical judgment in the present study. Patients with multiple etiological diagnoses (e.g., diffuse ARDS—superimposed gradient and dorsal collapse, PE patients with DLD or FLD) might have a potential impact on the results. Fourth, the thresholds of quadrant ventilation and perfusion distribution and the corresponding scores might not be optimal. Data percentile distributions in the control group and clinical simplicity were taken into consideration when the scoring system was defined. With the aim of obtaining a high diagnostic specificity, the value of the lowest 5th percentile of quadrant distributions in the control group was selected as the cutoff value for the abnormal distribution defect. The quadrant distribution is relative, so a low distribution in one quadrant is accompanied by an elevated distribution in the other quadrants. For simplicity, we only scored the quadrants with a decreasing distribution. A certain limitation of the proposed scoring system is that the sample size of control patients was small. Further studies are, therefore, required to validate or optimize the scoring system. Fifth, most patients were sedated and intubated in the present study. We found that it could be difficult in some awake ARF patients to perform a sufficient breath hold. Moreover, these patients normally generate respiratory muscular contractions that could modify the venous return and cardiac output. Hence, the saline-contrast EIT method might be more suitable for sedated patients under mechanical ventilation. Sixth, in the present study, lung perfusion was measured only at the end-expiration. Different airway pressures at end-inspiration and end-expiration could cause variation in lung perfusion. It is debatable whether lung perfusion by EIT should be measured at end-inspiration or end-expiration [46] or whether it matches average perfusion during normal tidal breathing. Inspiratory hold could be more readily implemented for patients with mild sedation, but a higher airway pressure at end-inspiration may cause an impairment of global circulation. The following potential benefits of lung perfusion at end-expiration were identified: (1) little impact on venous return and circulation and (2) a significant change in chest impedance might be easier caused by saline bolus at lower global impedance of end-expiration. In some preliminary data, we did not find differences in perfusion when obtained during a short interruption of ventilation at end-expiration and end-inspiration (the difference in global inhomogeneity index for perfusion was < 10%). We suspected that small tidal volume did not have a pronounced influence on regional lung perfusion. Further studies are required to compare the clinical relevance of end-inspiration occlusion and end-expiration methods.

The extensive limitations might limit the clinical applicability of the study. Advanced mathematical analysis and machine learning maybe helpful to explore the topic provided more patient data.

## Conclusions

In summary, the combined measurement of ventilation and perfusion by EIT with saline injection could identify probable etiologies of ARF at bedside. The phenotype of EIT ventilation and perfusion imaging might be helpful for a broad diagnosis of ARF etiologies, and further study is required to validate clinical applicability of this method.

## Supplementary Information

**Additional file 1: Table S1.** Diagnoses and the used diagnostic methods for the three broad ARF etiologies. **Table S2.** Comparison of ARF group and control group.

## Data Availability

Not applicable.

## Abbreviations

AUC: Areas under the receiver operating characteristic curve
ARF: Acute respiratory failure
APCHEII: Acute Physiology and Chronic Health Evaluation II score
DLD: Diffuse lung disease
EIT: Electrical impedance tomography
FLD: Focal lung disease
PE: Pulmonary embolism
LL: Lower left
LR: Lower right
UL: Upper left
UR: Upper right
