# Supplementary material for: Three broad classifications of acute respiratory failure etiologies based on regional ventilation and perfusion by electrical impedance tomography: a hypothesis-generating study
Source: Ann Intensive Care. 2021 Aug 28;11:134. doi: 10.1186/s13613-021-00921-6 (PMC8401348; doi:10.1186/s13613-021-00921-6)
Supplement: Supplementary file 1 — Additional file 1: Table S1. Diagnoses and the used diagnostic methods for the three broad ARF etiologies. Table S2. Comparison of ARF group and control group. [file 13613_2021_921_MOESM1_ESM.docx]

**Three broad classifications of acute respiratory failure etiologies based on regional ventilation and perfusion** **by electrical impedance tomography**

**Table S1 —****Diagnoses and the used diagnostic Methods for the three broad ARF etiologies**

| Diagnoses | Method |
| --- | --- |
| For all patients in study group | History, clinical examination, radiography (chest X-ray, CT and CTPA examination) evaluated by radiologists, bedside lung-heart ultrasound performed by trained ICU physicians with certification, favorable clinical progression under treatment, and: CT when available |
| Pulmonary embolism related disease group | Embolisms in deep vein and/or right atrium  Echocardiography (signs of acute cor pulmonale: acute enlargement of right heart, septal flattening, fixed inferior vena cava, etc.) or/and CTPA |
| Diffuse lung involvement disease | Diffuse interstitial syndrome diagnosed by ultrasound/CT  Ground glass opacity diagnosed by CT  Lung edema caused by fluid overload (diffuse “B” line in the anterior and lateral chest zone, fixed inferior vena cava or/and high CVP, negative fluid balance improve the oxygenation) |
| Focal lung involvement disease | Unilateral focus atelectasis/pneumonia/consolidation diagnosed by CT and/or bedside ultrasound.  Lateral gravity-dependent consolidation by CT and/or ultrasound (A line in the anterior zone and consolidation sign of tissue-like echotexture in the lateral/posterior chest)  Pneumothorax, identified by X ray and ultrasound and/or CT if necessary (bilateral and/or unilateral)  Pleural effusion, identified by ultrasound or/and CT (bilateral and/or unilateral ) |

**Table S2. Comparison of ARF group and control group**

| Variables | Control group  n=15 | ARF group  n=93 | P-value |
| --- | --- | --- | --- |
| Age(years)  Sex (female/male)  APACHE II score | 54±14  6/9  14±5 | 62±15  40/53  20±8 | 0.040  0.95  0.005 |
| HR (bpm)  MAP (mmHg)  FiO_2_ (%)  PaO_2_/FiO_2_ | 78(65, 93)  87±9  30(30, 35)  390(347, 474) | 92±16  84±11  46±17  210(152, 276) | 0.012  0.168  <0.0001  <0.0001 |
| UR-Vent (%)  UL-Vent (%)  LR-Vent (%)  LL-Vent (%)  UR-Perf (%)  UL-Perf (%)  LR-Perf (%)  LL-Perf (%)  UR- V(%)/Q(%)  UL- V(%)/Q(%)  LR- V(%)/Q(%)  LL- V(%)/Q(%)  *VQ Match_%_*  *DeadSpace_%_*  *Shunt_%_*  *Defect_V_* score  *Defect_Q_* score  *Defect_V+Q_* score | 31±7  29±9  22±10  17±6  24(19,30)  27±5  22(21,27)  22(20, 30)  1.3(1.1, 1.5)  1.1(0.8, 1.3)  0.9(0.7, 1.1)  0.6(0.7, 0.8)  72(65, 80)  12(7, 17)  12(8, 19)  0(0,1)  0(0, 0)  0(0, 1) | 30(26, 33)  31(21, 36)  21(12, 31)  18(13, 28)  27(17, 34)  26(21-32)  22(15-29)  23(18-32)  1.2(1.0, 1.7)  1.0(0.7, 1.3)  0.9(0.6, 1.2)  0.8(0.5, 1.0)  63(47, 72)  16(10, 28)  15(7, 29)  1(0, 3)  0(0, 1)  2(1, 4) | 0.594  0.439  0.548  0.729  0.845  0.539  0.436  0.993  0.979  0.480  0.880  0.594  0.002  0.05  0.363  0.003  0.01  <0.0001 |
| 28day mortality | 0/15 | 18/93 | 0.07 |

APACHE, Acute Physiology and Chronic Health Evaluation; HR, heart rate(bpm); MAP, mean arterial pressure (mmHg); PaO_2_: arterial partial pressure of oxygen; FiO_2_: fractional inspired oxygen concentration; NE, norepinephrine (μg/kg.min);

UR, upper right; UL, upper left; LR, lower right; LL, lower left; Pts, patients; NE, norepinephrine (μg/kg.min); V(%)/Q(%), relative regional ventilation(%)/corresponding regional perfusion(%); *Defect_Q_* perfusion defect score; *Defect_V_* ventilation defect score; The values are presented either as mean±SD or as median (25^th^ 75^th^ percentiles).
